# Comparative metabolic profiling, enzyme inhibitory activities, and *in-silico* analysis of the hexane extract and the hydrodistilled oil of *Boswellia serrata*

**DOI:** 10.1371/journal.pone.0348178

**Published:** 2026-05-08

**Authors:** Heba A. S. El-Nashar, Esraa A. Elhawary, Nilofar Nilofar, Mahmoud A. El Hassab, Taghreed A. Majrashi, Wagdy Eldehna, Gokhan Zengin, Omayma A. Eldahshan

**Affiliations:** 1 Department of Pharmacognosy, Faculty of Pharmacy, Ain Shams University, Cairo, Egypt; 2 Department of Pharmacognosy, Faculty of Pharmacy, Modern University for Technology & Information, Cairo, Egypt; 3 Department of Biology, Science Faculty, Selcuk University, Campus, Konya, Turkey; 4 Department of Pharmacy, Botanic Garden “Giardino dei Semplici”, Università degli Studi “Gabriele d’Annunzio”, Chieti, Italy; 5 Department of Medicinal Chemistry, Faculty of Pharmacy, King Salman International University (KSIU), South Sinai, Egypt; 6 Department of Pharmacognosy, College of Pharmacy, King Khalid University, Abha, Asir, Saudi Arabia; 7 Department of Pharmaceutical Chemistry, Faculty of Pharmacy, Kafrelsheikh University, Kafrelsheikh, Egypt; 8 Department of Pharmaceutical Chemistry, Faculty of Pharmacy, Pharos University in Alexandria, Alexandria, Egypt; Council of Scientific & Industrial Research Indian Institute of Integrative Medicine, INDIA

## Abstract

Frankincense (*Boswellia* spp.) oleogum resin is a valuable natural source of bioactive phytoconstituents with diverse therapeutic potential. In this study, the hydrodistilled essential oil (EO) and *n*-hexane extract (HE) of *Boswellia serrata* gums were analyzed through gas chromatography–mass spectrometry (GC-MS) to determine their phytochemical composition. The GC-MS results in the identification of 62 and 71 components in the EO and HE, respectively. Acetic acid octyl ester (41.09%) and nerolidol (13.64%), were the major components of the EO. Meanwhile, incensole (28.56%), (1S,2E,4S,5R,7E,11E)-cembra-2,7,11-trien-4,5-diol (13.54%), and 24-norursa-3,12-diene (9.25%) in the HE. Regarding the antioxidant effects, the EO exhibited significantly higher antioxidant capacity compared to the HE (DPPH: 9.24 and 6.50 mg TE/g; ABTS: 25.71 and 4.94 mg TE/g), respectively. Moreover, the EO was more potent in the CUPRAC test (61.12 mg TE/g for the essential oil and 50.62 mg TE/g for HE), while the *n*-hexane extract (72.68 mg TE/g) showed stronger ability than the EO (13.22 mg TE/g) in the FRAP assay. The EO had a higher ability in phosphomolybdenum and metal chelation tests in comparison with the HE extracts. Further, the oil showed more potent inhibitory activity against cholinesterase, *α*-glucosidase, and tyrosinase than the HE extract. The HE extract was only more active on *α*-amylase compared to the oil. These findings suggest that olibanum EO possesses potent bioactive compounds that may contribute to the management of oxidative stress and age-related conditions, including Alzheimer’s disease, diabetes mellitus, and skin hyperpigmentation.

## 1. Introduction

In recent years, plant-derived materials have attracted much consideration and interest [1–5]. Valuable plant phytoconstituents have become advantageous when used to obtain a variety of promising biological effects [6].

The *Boswellia* genus encompasses about 20 species present in dry climates from West Africa, Arabia, to Tanzania. Additionally, Madagascar and India include some species. The genus is endemic in Africa (75% of the species) in the northeast regions.

*Boswellia* plants, Burseraceae, represent a rich source of plant phytoconstituents. *Boswellia serrata* is a famous species and is known as Frankincense or olibanum (In Arabic: Liban Dakar) [7,8]. *Boswellia serrata* is predominantly present in India, thriving in the Madhya Pradesh forests, Rajasthan, Gujarat, Assam, Bihar, and Odisha. Historically, various old civilizations, including the Persians, the Chinese, and those from early American societies, utilized olibanum for medicinal purposes.

The plant is a deciduous tree that is popular due to its oleogum resin, which carries a lot of beneficial effects in many diseases. This resinous material can be easily extracted by non-polar solvents as hexane and petroleum ether. The oleogum resin has been found to have beneficial effects on many diseases, such as asthma, bronchitis, cough, dysentery, diarrhea, cardiovascular diseases, boils, ringworms, fevers, mouth sores, skin diseases, and vaginal secretions. The volatile components identified in the resin include geraniol, eleneol, cadinene, *β*-pinene, linalool, phenols, bornyl acetate, terpenyl acetate, serratol, *α*/*β*-amyrin, and boswellic acid. Icensole is the main key ingredient from the hydrodistilled oil and hexane extract of the oleogum resin, with a quantity reaching 75% [7–9]. It was found to exhibit a plethora of *in vitro* biological activities like antimicrobial activity [10], anticancer activity [11], antioxidant activity [12], anti-inflammatory activity [13], antioxidant [14], and antimutagenic [15]. In addition, it showed different in vivo activities including anti-Alzheimer’s disease [16–20], anti-Parkinson’s disease [21], antioxidant, immunomodulatory [22], anti-inflammatory [23], anticancer [24,25], analgesic [26], smooth muscle relaxant [26], antarthritic [27], diuretic [28], antidiarrheal [29], antiasthma [30] and anti-colitis [31].

Antioxidant activity of certain plants is usually attributed to their flavonoid and phenolic contents in addition to the presence of abundant oxygenated hydrocarbons in their essential oils or non-polar extracts using petroleum ether or *n*-hexane. The ability of certain plant extracts or volatile components to inhibit certain key biological enzymes, such as acetylcholinesterase, butylcholinesterase, *α*-glucosidase, and α-amylase, is of valuable medical importance [32–34]. Thus, this study was designed to perform a comparative study between the oil and hexane extract of *Boswellia serrata* gums *via* GC/MS chemical analysis, besides investigation of the antioxidant properties and inhibitory effect against the above-mentioned enzymes participating in skin hyperpigmentation, Alzheimer’s, diabetes mellitus.

## 2. Materials and methods

### 2.1 Plant material, essential oil isolation and extract preparation

The gums of *Boswellia serrata* (Burseraceae) were obtained in February 2023 from the local market, Cairo Governorate, Egypt. It was deposited as a voucher specimen (PHG-P-BS-479), at the Pharmacognosy Department, School of Pharmacy (Ain Shams University), Cairo, Egypt. The Clevenger apparatus was used for hydrodistillation of 500g of the gums (5 hrs.). The oil was preserved at −4ºC for the chemical and biological assessments.

About 200g of *B. serrata* gums were soaked in *n*-hexane (2L) for three days, and the extraction was repeated 3 times. The extract was completely evaporated by vacuum at 45 °C to yield 6.30 g. The obtained extract was kept and stored at −4ºC.

### 2.2 Gas Chromatography/Mass Spectrometry (GC/MS) analysis

The GC/MS analysis of the oil and n-hexane extract from the gums was conducted using a Shimadzu GC-MS-QP 2010 (Kyoto, Japan). The TRACE GC Ultra Gas Chromatograph is manufactured by Thermo Scientific Corporation, USA, equipped with a thermo-mass detector at the pharmacognosy department, Ain Shams University, Cairo, Egypt. The GC/MS system utilized a high-performance TG-5MS capillary column from Restek, USA, measuring 30 m in length and 0.25 mm in diameter, with a precise 0.25 *μ*m film thickness, ensuring exceptional sensitivity and resolution. The capillary column was linked directly to a quadrupole mass spectrometer (SSQ 7000; Thermo-Finnigan). For the analysis, a 1% v/v diluted sample (1 *µL* injection volume) was used, with helium as the carrier gas at a steady. The rate of flow is established at 1.0 mL/min and 1:15 as a split ratio. The oven temperature begins at 80°C for 2 minutes (isothermal), then increases at a rate of 5.0°C per minute until it reaches 300°C (programmed). This temperature is held for an additional 5 minutes (isothermal). The temperatures of both the injector and detector were kept at 280°C. The mass spectra were obtained with the following settings: interface temperature at 280°C, ion source temperature at 200°C, and electron ionization (EI) mode at 70 eV, using a scan spectral range from m/z 35–500. The relative proportions of the *n*-hexane extract constituents were expressed as percentages based on peak area normalization.

### 2.3 GC/MS identification of chemical components of the hydrodistilled oil and the *n*-hexane extract of *Boswellia serrata* gums

The volatile compounds were initially identified by comparing their spectra of GCMS, patterns of fragmentation, and Kovats indices with the NIST and Wiley libraries, as well as existing literature. [35–41]. The retention indices were calculated using a series of homologous *n*-alkanes ranging from C8 to C28. that were injected under the same conditions. The percentage of the peak area of each compound was determined relative to the percentage of the total area of the entire chromatogram of FID (100%).

### 2.4 Antioxidant and enzyme inhibitory assays

To evaluate essential oil and hexane antioxidant activity, six different spectrophotometric tests were carried out. They are the ABTS (2,2’-azino-*bis* (3-ethylbenzothiazoline-6-sulfonic acid) and DPPH (2,2-diphenyl-1-picrylhydrazyl) assays, which assess the ability of antioxidants to neutralize free radicals. The reduction activity of the extract was evaluated by FRAP, the Ferric Reducing Ability of Plasma and CUPRAC, and Cupric Ion Reducing Antioxidant Capacity tests. Additionally, the Phosphomolybdenum and Ferrozine assays measured the total antioxidant capacity and metal chelating potential, respectively. Except for Metal-chelating Activity (MCA), each assay was evaluated using a Trolox standard, while MCA was compared using ethylenediamine tetraacetic acid (EDTA) equivalents per gram of essential oil/extract. Detailed steps can be found in our former works [42,43]. To evaluate the inhibitory potential of the extracts on different enzymes, we conducted assays for AChE, BChE, tyrosinase, amylase, and β-glucosidase. Experimental procedures are available in our earlier publications [42,44]. AChE and BChE inhibition were measured as mg of galanthamine equivalents (GALAE)/g of hydrodistilled oil or extract. Tyrosinase inhibition was expressed in mg of kojic acid equivalents (KAE)/g of hydrodistilled oil/extract, and *α*-amylase inhibition was quantified in mmol of acarbose equivalents (ACAE) per g of essential oil/extract.

### 2.5 *In silico* molecular docking study

The X-ray 3D structures of NADPH oxidase, butyrylcholinesterase, tyrosinase, α-amylase, and α-glucosidase were obtained from the Protein Data Bank with the following IDs: 2cdu (resolution 1.80 Å), 6esj (resolution 2.98 Å), 5m8q (resolution 2.85 Å), 4gqq (resolution 1.35 Å), and 3wy2 (resolution 1.47 Å), respectively. Vina Autodock and MGL tools were used to perform the docking studies [45,46]. The major components of the essential oil, acetic acid octyl ester, nerolidol, and caprylic alcohol, alongside the *n*-hexane, extracted major components, incensole, (1S,2E,4S,5R,7E,11E)-cembra-2,7,11-trien-4,5-diol, and 24-norursa-3,12-diene were implemented in the docking study. All five receptors and the six compounds were converted to pdbqt format using MGL tools, as required by Vina Autodock^®^. The active site of each target was identified based on the binding of the respective co-crystallized ligand, with the following dimensions 22*22*22 Å in the x, y and z directions. The docking results were then analyzed using the Discovery Studio visualizer, which also generated 2D interaction diagrams.

## 3. Results

### 3.1 Identification of metabolites through GC/MS analysis of *Boswellia serrata* gums

**Fig 1:**
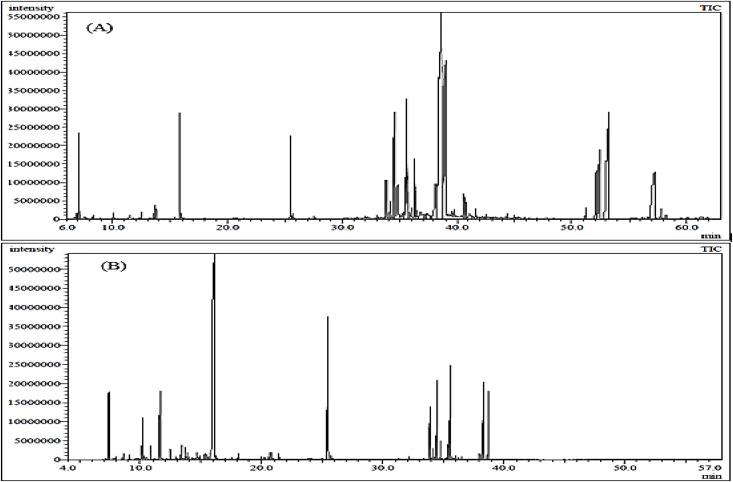
GC-MS chromatogram of the hydrodistilled oil (A) and *n*-hexane extract (B) of *Boswellia serrata* gum.

**Fig 2:**
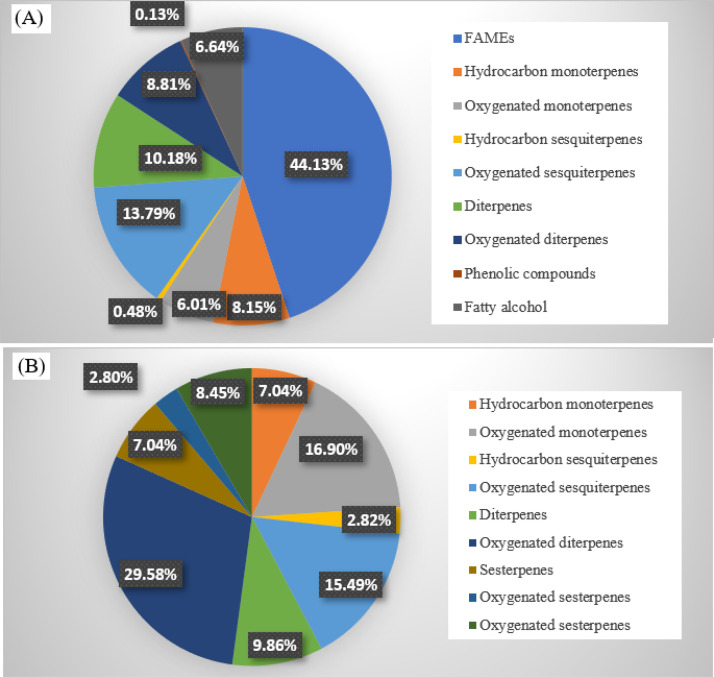
Pie charts display the distribution of different classes of compounds (%) in the essential oil (A) and *n*-hexane extract (B) of *Boswellia serrata* gums.

The results of the analysis of both the essential oil and the *n*-hexane extract from *Boswellia serrata* gums are provided in Fig 1 and Table 1. The GC/MS investigation results in the identification of 62 and 71 compounds in oil and extract, respectively. The identified compounds accounted for 98.32% and 99.20% of the oil and extract, respectively. The hydrodistilled oil has been predominated by fatty acid methyl esters (FAMEs; 44.13%), followed by oxygenated sesquiterpenes (13.79%), diterpenes (10.18%), oxygenated diterpenes (8.81%), hydrocarbon monoterpenes (8.15%) and oxygenated monoterpene (6.01%) as represented in Fig 2A. While the *n*-hexane extract elicited oxygenated diterpenes as the major class of compounds followed by the oxygenated monoterpenes and oxygenated sesquiterpenes (Fig 2B).

**Table 1:** GC-MS Chemical composition (%) of the essential oil and *n*-hexane extract of *Boswellia serrata* gum.

No.	Compound name	Retention time (t_R_)	Molecular formula	Kovats index (KI)		Peak area (%)	
				KI_Exp._	KI_Rep._	Essential oil	*n*-Hexane extract
1.	*α*-Thujene	7.15	C_10_H_16_	909	905	0.07	0.15
2.	*α*-Pinene	7.41	C_10_H_16_	919	925	4.41	2.04
3.	Thuja-2,4(10)-diene	8.320	C_10_H_14_	958	961	–	0.16
4.	Sabinene	8.50	C_10_H_16_	958	964	0.07	–
5.	Dehydrosabinene	8.59	C_10_H_14_	961	960	0.18	–
6.	*β*-Pinene	9.05	C_10_H_16_	978	980	0.18	–
7.	*β*-Myrcene	9.46	C_10_H_16_	993	992	0.03	0.04
8.	3-Carene	9.61	C_10_H_16_	999	1001	0.13	–
9.	Acetic acid, hexyl ester	9.78	C_8_H_16_O_2_	1004	1006	0.10	–
10.	*p*-Cymene	10.08	C_10_H_14_	1014	1015	0.53	–
11.	D-Limonene	10.22	C_10_H_16_	1028	1028	1.90	0.16
12.	(*E*)-*β*-Ocimene	10.82	C_10_H_16_	1038	1037	0.46	–
13.	*γ*-Terpinene	11.14	C_10_H_16_	1048	1050	0.05	–
14.	Caprylic alcohol	11.69	C_8_H_18_O	1066	1068	6.64	–
15.	*p*-Cymenene	12.14	C_10_H_12_	1080	1080	0.14	–
16.	*β*-Linalool	12.47	C_10_H_18_O	1091	1092	0.67	0.04
17.	dehydrolinalool	12.67	C_10_H_16_O	1097	1090	0.06	0.17
18.	*α*-Thujone	1295	C_10_H_16_O	1106	1106	0.11	0.02
19.	*α*-Campholenal	13.25	C_10_H_16_O	1116	1117	0.19	0.05
20.	(*E*)-Thujone	13.40	C_10_H_16_O	1121	1114	0.65	0.03
21.	2(10)-Pinen-3-ol	13.68	C_10_H_16_O	1131	1134	0.83	–
22.	(*E*)-Pinocarveol	13.88	C_10_H_16_O	1136	1138	0.44	0.30
23.	(*E*)-Sabinol	14.02	C_10_H_16_O	1141	1140	0.22	–
24.	Bicyclo[3.1.1]heptan-3-one, 2,6,6-trimethyl-	14.32	C_10_H_16_O	1150	1160	0.07	–
25.	Pinocarvone	14.39	C_10_H_14_O	1152	1158	0.07	0.06
26.	p-Mentha-1,5-dien-8-ol	14.65	C_10_H_16_O	1161	1159	0.49	–
27.	Terpinen-4-ol	14.88	C_10_H_18_O	1168	1171	0.25	–
28.	*m*-Cymen-8-ol	15.31	C_10_H_14_O	1182	1183	0.23	–
29.	Thuj-3-en-10-al	15.390	C_10_H_14_O	1186	1184	–	0.05
30.	*α*-Terpineol	15.41	C_10_H_18_O	1185	1188	0.48	–
31.	Myrtenol	15.60	C_10_H_16_O	1192	1193	0.16	–
32.	Acetic acid, octyl ester	16.09	C_10_H_20_O_2_	1208	1210	41.09	3.16
33.	(*Z*)-Carveol	16.28	C_10_H_16_O	1215	1217	0.11	0.04
34.	Carvone	16.94	C_10_H_14_O	1238	1240	0.03	–
35.	Orcinol dimethyl ether	17.55	C_9_H_12_O_2_	1259	1260	0.10	–
36.	Bornyl acetate	18.06	C_12_H_20_O_2_	1277	1277	0.25	–
37.	Thymol	18.48	C_10_H_14_O	1291	1292	0.02	–
38.	Citronellyl acetate	19.92	C_12_H_22_O_2_	1342	1341	0.30	–
39.	Copaene	20.59	C_15_H_24_	1365	1367	0.14	0.03
40.	Neryl acetate	20.76	C_12_H_20_O_2_	1371	1369	0.38	0.05
41.	(*Z*)-*β-*Elemene	21.03	C_15_H_24_	1381	1380	0.02	–
42.	Acetic acid, decyl ester	21.45	C_12_H_24_O_2_	1395	1394	0.32	–
43.	(*Z*)-Methyl eugenol	21.63	C_11_H_14_O_2_	1402	1401	0.03	–
44.	Caryophyllene	21.81	C_15_H_24_	1409	1409	0.01	–
45.	Selina-5,11-diene	22.43	C_15_H_24_	1433	1446	0.05	–
46.	Curcumene	23.44	C_15_H_22_	1472	1472	0.04	–
47.	Eudesma-4(14),11-diene	23.58	C_15_H_24_	1478	1479	0.15	–
48.	Bisabolene	24.09	C_15_H_24_	1498	1500	0.07	–
49.	Nerolidol	24.49	C_15_H_26_O	1555	1552	13.64	2.22
50.	Dodecanoic acid	25.72	C_12_H_24_O_2_	1561	1562	–	0.30
51.	Octanoic acid, hexyl ester	25.87	C_14_H_28_O_2_	1567	1571	0.03	–
52.	Junenol	27.16	C_15_H_26_O	1620	1619	–	0.03
53.	Dodecanoic acid, trimethylsilyl ester	27.56	C_15_H_32_O_2_Si	1637	1643	–	0.08
54.	Allohimachalol	28.11	C_15_H_26_O	1661	1662	–	0.02
55.	*cis*-Octadecanoic acid, (2-phenyl-1,3-dioxolan-4-yl) methyl ester	30.39	C_28_H_46_O_4_	1760	1761	–	0.09
56.	(*E*)-Isovalencenol	31.31	C_15_H_24_O	1800	1793	0.05	0.04
57.	Myristic acid, trimethylsilyl ester	32.15	C_17_H_36_O_2_Si	1840	1840	0.12	0.08
58.	Cubitene	33.06	C_20_H_32_	1884	1878	–	0.07
59.	*di-*hydro-Columellarin	33.43	C_15_H_22_O_2_	1901	1900	0.10	0.09
60.	Methyl hexadecanoate	33.55	C_17_H_34_O_2_	1907	1921	–	0.04
61.	Palmitic acid, methyl ester	33.86	C_17_H_34_O_2_	1922	1921	2.47	–
62.	Thunbergene	34.13	C_20_H_32_	1935	1939	0.55	4.53
63.	(3*E*)-Cembrene A	34.32	C_20_H_32_	1944	1948	–	0.10
64.	Cembrene A	34.50	C_20_H_32_	1953	1956	4.14	3.15
65.	5*α*-3-ethyl-3-hydroxy-Androstan-17-one	35.43	C_21_H_34_O_2_	1992	1992	0.76	0.85
66.	Kaur-16-ene	35.62	C_20_H_32_	2007	2007	5.11	–
67.	Verticilla-4(20),7,11-triene	35.77	C_20_H_32_	2016	2026	0.38	5.79
68.	Manool	36.49	C_20_H_34_O	2055	2057	0.16	0.37
69.	Pentacyclo[9.1.0.0(2,4).0(5,7).0(8,10)]dodecane, 3,3,6,6,9,9,12,12-octamethyl-, anti,syn	36.86	C_20_H_32_	2075	2076	–	0.66
70.	Abietadiene	37.09	C_20_H_32_	2088	2087	–	0.26
71.	Viridiflorol	37.30	C_15_H_26_O	2099	2098	–	0.37
72.	Laurenan-2-one	37.43	C_20_H_32_O	2107	2116	0.03	0.39
73.	(*E*)-Isoeugenyl benzyl ether	37.73	C_17_H_18_O_2_	2123	2125	–	0.22
74.	Cembrenol	38.11	C_20_H_34_O	2144	2161	–	3.38
75.	Incensole	38.32	C_20_H_34_O_2_	2156	2159	4.72	28.56
76.	(1*S*,2*E*,4*S*,5*R*,7*E*,11*E*)-Cembra-2,7,11-trien-4,5-diol	38.72	C_20_H_34_O_2_	2187	2188	3.12	13.54
77.	Ugandensidial	39.07	C_17_H_24_O_5_	2196	2198	–	0.01
78.	Octadecanol acetate	39.13	C_20_H_40_O_2_	2200	2209	–	0.04
79.	(*E*)-Phytol acetate	39.39	C_22_H_42_O_2_	2214	2218	–	0.07
80.	Sclareol	39.53	C_20_H_36_O_2_	2222	2223	–	0.24
81.	7-*α*-hydroxy-Manool	39.81	C_20_H_34_O_2_	2237	2235	–	0.35
82.	1-Heptatriacotanol	39.95	C_37_H_76_O	2245	2245	–	0.08
83.	Icensole oxide	40.67	C_20_H_34_O_3_	2285	2280	0.02	1.37
84.	Nerolidol isobutyrate	41.07	C_19_H_32_	2306	2305	–	0.85
85.	4-*epi*-Abietol	41.91	C_20_H_32_O	2352	2344	–	0.07
86.	Communic acid	42.24	C_20_H_30_O_2_	2370	2366	–	0.20
87.	Labd-7,13-dien-15-ol acetate	42.51	C_22_H_36_O_2_	2385	2392	–	0.18
88.	17-(acetyloxy)-(4*β*)-Kauran-18-al	42.76	C_22_H_34_O_3_	2399	2407	–	0.19
89.	Labd-(13*E*)-8,15-diol	43.11	C_20_H_36_O_2_	2421	2422	–	0.09
90.	6-keto-Ferruginol	43.57	C_20_H_28_O_2_	2451	2457	–	0.12
91.	2-Methyltetracosane	43.88	C_25_H_52_	2470	2465	–	0.08
92.	Methyl 7,13,15-abietatrienoate	44.35	C_21_H_30_O_2_	2500	2501	–	0.27
93.	Hinokione	44.99	C_20_H_28_O_2_	2542	2550	–	0.13
94.	Hinokienone	45.32	C_20_H_26_O_2_	2563	2557	–	0.05
95.	Hexacosane	45.89	C_26_H_54_	2600	2600	–	0.04
96.	24-Norursa-3,9(11),12-triene	52.11	C_29_H_44_	2999	3042	–	3.02
97.	24-Noroleana-3,12-diene	52.40	C_29_H_46_	3018	3057	–	3.85
98.	1-Heptatriacotanol	52.61	C_37_H_76_O	3031	3031	–	0.02
99.	24-Norursa-3,12-diene	53.16	C_29_H_46_	3067	3067	–	9.25
100.	(3*β*)-9,19-Cyclolanost-24-en-3-ol	55.24	C_30_H_50_O	3200	3200	–	0.05
101.	Lanosterol	55.61	C_30_H_50_O	3224	3224	–	0.23
102.	24-Norursa-3,12-dien-11-one	57.20	C_29_H_44_O	3326	3352	–	5.55
103.	Betulinaldehyde	59.53	C_30_H_48_O	3476	3477	–	0.69
104.	*α*-Amyrin	60.13	C_30_H_50_O	3514	3513	–	0.14
105.	24-Noroleana-3,12-diene	62.17	C_29_H_46_	3646	3645	–	0.02
	**Total identified**					**98.32%**	**99.05%**
	Fatty acid methyl esters (FAMEs)					44.13%	–
	Hydrocarbon monoterpenes					8.15%	7.04%
	Oxygenated monoterpenes					6.01%	16.90%
	Hydrocarbon sesquiterpenes					0.48%	2.82%
	Oxygenated sesquiterpenes					13.79%	15.49%
	Hydrocarbon diterpenes					10.18%	9.86%
	Oxygenated diterpenes					8.81%	29.58%
	Phenolic compounds					0.13%	–
	Fatty alcohol					6.64%	–
	Hydrocarbon sesterpenes					–	7.04%
	Oxygenated sesterpenes					–	2.82%
	Oxygenated triterpenes					–	8.45%

**Fig 3:**
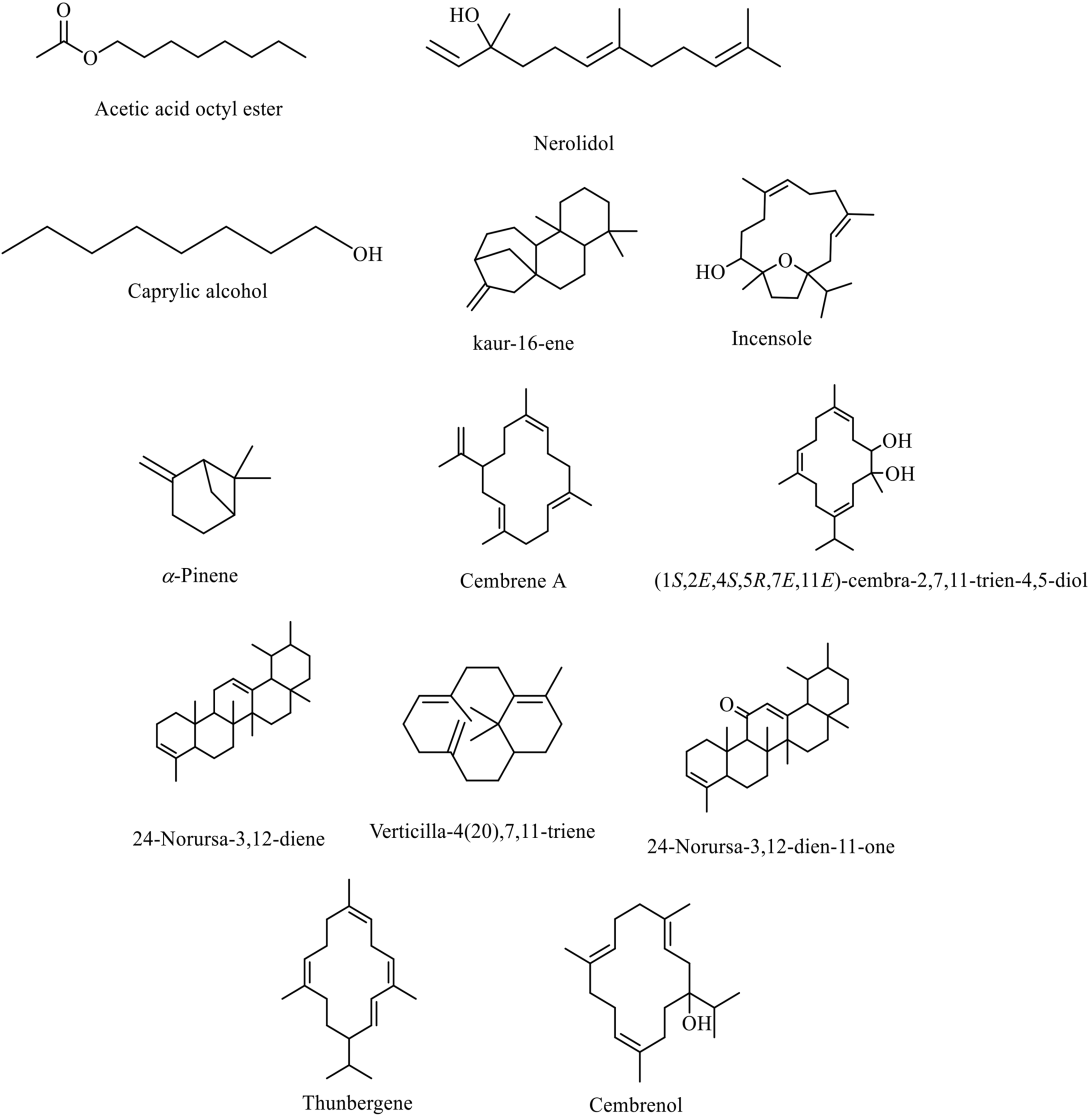
The chemical structures of the major volatile compounds detected in the essential oil and *n*-hexane extract of *Boswellia serrata* gum.

In the essential oil, Acetic acid octyl ester (41.09%) and nerolidol (13.64%) showed the highest dominance, followed by caprylic alcohol (6.64%), kaur-16-ene (5.11%), incensole (4.72%), *α*-pinene (4.41%), and cembrene A (4.14%). Regarding the n-hexane extract, the most abundant volatile components were incensole (28.56%), (1S,2E,4S,5R,7E,11E)-cembra-2,7,11-trien-4,5-diol (13.54%), 24-norursa-3,12-diene (9.25%), verticilla-4(20),7,11-triene (5.79%), 24-norursa-3,12-dien-11-one (5.55%), thunbergene (4.53%), cembrenol (3.38%), acetic acid octyl ester (3.16%) and cembrene A (3.15%). As abovementioned, the oxygenated diterpenoids were the most abundant class of volatile components identified from *B. serrata*. Oxygenated diterpenoids came first with incensole (28.56%) and cembrenol (3.38%) as the main components identified from this class. Moreover, oxygenated monoterpenoids showed the second most identified class with their main compounds such as *trans*-pinocarveol (0.30%) and dehydro-linalool (0.17%). The third most abundant class was the oxygenated sesquiterpenes, which were mainly represented by nerolidol (2.22%) and viridiflorol (0.37%). The major compound structures detected in the hydrodistilled oil and *n*-hexane extract of *Boswellia serrata* gums are cumulatively shown in Fig 3

### 3.2 Antioxidant activity of the essential oil and the *n*-hexane extract of Olibanum

The antioxidant characteristics of the volatile oil and n-hexane extract were evaluated using various assays, including reducing power (FRAP and CUPRAC), radical quenching (ABTS and DPPH), metal chelation, and phosphomolybdenum. The results are presented in Table 2. In both radical scavenging assays, the essential oil (DPPH: 9.24 mg TE/g; ABTS: 25.71 mg TE/g) exhibited higher activity than the n-hexane extract (DPPH: 6.50 mg TE/g; ABTS: 4.94 mg TE/g). Furthermore, the essential oil (61.12 mg TE/g) showed greater performance in the CUPRAC assay compared to the *n*-hexane extract (50.62 mg TE/g), while the n-hexane extract (72.68 mg TE/g) demonstrated a stronger ability than the essential oil (13.22 mg TE/g) in the FRAP assay. The essential oil also had a higher ability in phosphomolybdenum and metal chelation tests compared to the *n*-hexane extract.

**Table 2:** Antioxidant effects of the essential oil and *n*-hexane extracts.

Samples	DPPH (mg TE/g)	ABTS (mg TE/g)	CUPRAC (mg TE/g)	FRAP (mg TE/g)	PBD (mmol TE/g)	MCA (mg EDTAE/g)
Essential oil	9.24 ± 0.49	25.71 ± 0.64	61.12 ± 4.48	13.22 ± 0.44	11.51 ± 0.18	32.09 ± 0.67
*n*-Hexane extract	6.50 ± 0.51	4.94 ± 0.24	50.62 ± 1.66	72.68 ± 0.87	2.20 ± 0.15	18.01 ± 0.90

*Values are reported as mean ±SD of three parallel experiments. TE: Trolox equivalent; EDTAE: EDTA equivalent.

### 3.3 The enzyme inhibitory potential of the essential oil and *n*-hexane extract of Olibanum

The enzyme inhibitory effects of the essential oil and *n*-hexane of Olibanum were investigated against several enzymes, including cholinesterase, *α*-glucosidase, *α*-amylase, and tyrosinase. The results are presented in Table 3. Except for *α*-amylase inhibition, in all other enzyme assays, the essential oil demonstrated a stronger effect compared to others. The *n*-hexane extract was only more active on *α*-amylase, compared to the essential oil.

**Table 3:** Enzyme inhibitory effects of essential oil and *n*-hexane extracts.

Samples	AChE (mg GALAE/g)	BChE (mg GALAE/g)	Tyrosinase (mg KAE/g)	*α*-Amylase (mmol ACAE/g)	*α*-Glucosidase (mmol ACAE/g)
Essential oil	2.14 ± 0.25	3.72 ± 0.14	43.31 ± 0.05	0.07 ± 0.01	2.56 ± 0.07
*n*-Hexane extract	1.98 ± 0.38	2.11 ± 0.04	38.05 ± 4.92	0.44 ± 0.01	2.54 ± 0.01

*Values are reported as mean ±SD of three parallel experiments. GALAE: Galanthamine equivalent; KAE: Kojic acid equivalent; ACAE: Acarbose equivalent

### 3.4 *In silico* molecular docking study

The primary components of the essential oil, namely acetic acid octyl ester, nerolidol, and caprylic alcohol, along with the main compounds of the *n*-hexane extract, such as incensole, (1*S*,2*E*,4*S*,5*R*,7*E*,11*E*)-cembra-2,7,11-trien-4,5-diol, and 24-norursa-3,12-diene, were docked into the active sites of the five enzymes: NADPH oxidase, butyrylcholinesterase, tyrosinase, *α*-amylase, and *α*-glucosidase. Those six compounds were selected as they represent the highest percentage in the major extract. As indicated in Table 4, all compounds revealed good binding scores on docking with these five targets.

**Table 4:** The docking scores for the main active components of the essential oil and *n*-hexane extract of olibanum.

	2cdu NADPH oxidase,	6esj Butyrylcholinesterase	5m8q tyrosinase,	4gqq *α*-amylase	3wy2 *α*-glucosidase
**Acetic acid octyl ester**	−8.4	−8.2	−11.9	−5.6	−10.2
**Nerolidol**	−7.5	−10.6	−7.6	−6.7	−12.3
**Caprylic alcohol**	−7.1	−8.5	−6.5	−5.8	−8.8
**Incensole**	−7.5	−10.5	−5.5	−5.4	−7.9
**1*S*,2*E*,4*S*,5*R*,7*E*,11*E*)-Cembra-2,7,11-trien-4,5-diol**	−7.3	9.2	−8.7	−6.1	−10.2
**24-Norursa-3,12-diene**	−9.4	8.1	−7.2	−7.1	−10.1
**X-ray reference**	−7.5	−9.9	−7.8	−7.6	−8.2
**RMSD values between redocked poses and X-rayreference**	0.88	0.64	0.67	0.84	0.94

**Fig 4:**
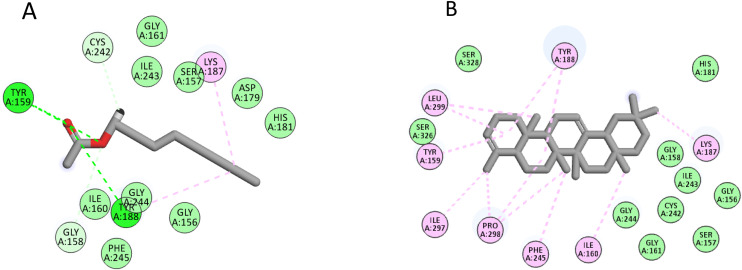
The docking of (A) acetic acid octyl, (B) 24-norursa-3,12-diene in the active site of NADPH oxidase enzyme (PDB code: 2cdu).

For the NADPH oxidase, the six compounds achieved docking scores from −7.1 to −9.4 Kcal/mol, where acetic acid octyl ester and 24-norursa-3,12-diene were the best compounds, achieving scores of −8.4 and −9.4 Kcal/mol, respectively. Inspecting Fig 4., Acetic acid octyl interacted with Tyr159 and Tyr188 through hydrogen bond interactions and with Lys187 and Tyr188, through hydrophobic interactions, while 24-norursa-3,12-diene formed only hydrophobic interactions with Tyr159, Ile160, Lys187, Tyr188, Phe245, Ille297, Pro298, and Leu299.

**Fig 5:**
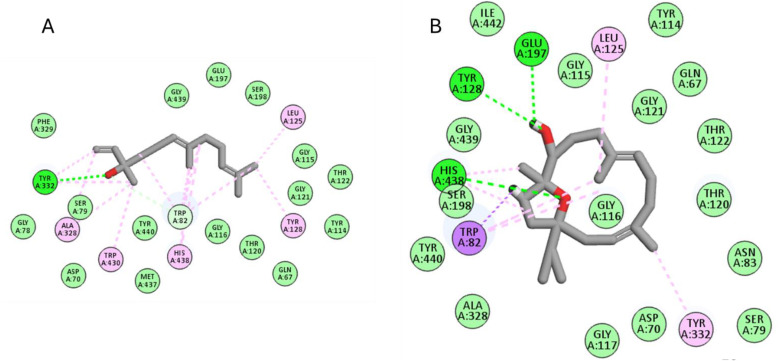
The docking of (A) nerolidol, (B) incensole in the active site of BChE enzyme (PDB code: 6esj).

For the BChE enzyme, the six compounds achieved docking scores from −8.1 to −10.6 Kcal/mol, where nerolidol and incensole were the best compounds, achieving scores of −10.6 and −10.5 Kcal/mol, respectively. As seen in Fig 5, nerolidol formed several hydrophobic interactions with Trp82, Leu125, Tyr128, Ala328, Tyr332, Trp430, His438, and one hydrogen bond with Tyr332. Similarly, incensole formed hydrophobic interactions with Trp82, Leu125, Tyr332, His438, and hydrogen bond interactions with Tyr128, Glu197, and His438.

**Fig 6:**
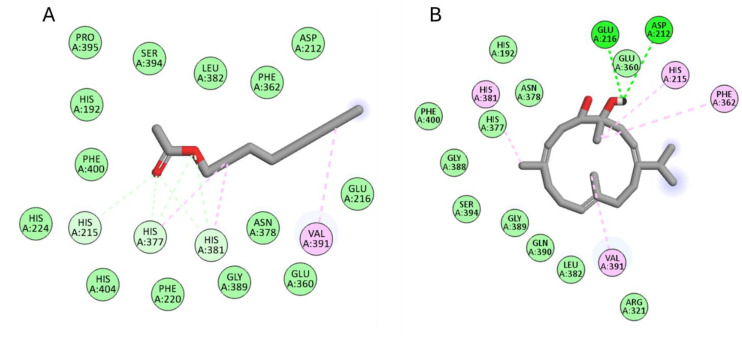
The docking of (A) Acetic acid octyl, (B) (1*S*,2*E*,4*S*,5*R*,7*E*,11*E*)-cembra-2,7,11-trien-4,5-diol in the active site of tyrosinase enzyme (PDB code: 5m8q).

The six compounds achieved docking scores ranging from −5.5 to −11.9 Kcal/mol against the tyrosinase enzyme. Acetic acid octyl ester and (1*S*,2*E*,4*S*,5*R*,7*E*,11*E*)-cembra-2,7,11-trien-4,5-diol achieved the best scores −11.9 and −8.7 Kcal/mol, respectively. As Fig 6 revealed, acetic acid octyl ester formed several interactions with His215, His377, His381, and Val391. Likewise, (1*S*,2*E*,4*S*,5*R*,7*E*,11*E*)-cembra-2,7,11-trien-4,5-diol interacted Asp212, His215, Glu216, Phe362, His381 and Val391.

**Fig 7:**
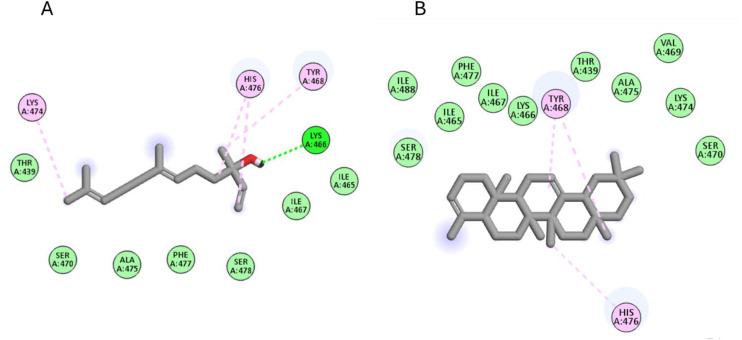
The docking of (A) nerolidol, (B) 24-norursa-3,12-diene, in the active site of *α*-amylase enzyme (PDB code: 4gqq).

In the docking with *α*-amylase, the major compounds achieved good scores ranging from −5.4 to −7.1 Kcal/mol. Amongst nerolidol and 24-norursa-3,12-diene were the best compounds with scorers −6.7 and −7.1 Kcal/mol, respectively. As seen in Fig 7, nerolidol interacted with Lys466, Tyr468, Lys474, and His476 through both hydrophobic and hydrogen bond interactions. On the other hand, 24-norursa-3,12-diene formed only hydrophobic interactions with Tyr468 and His476.

**Fig 8:**
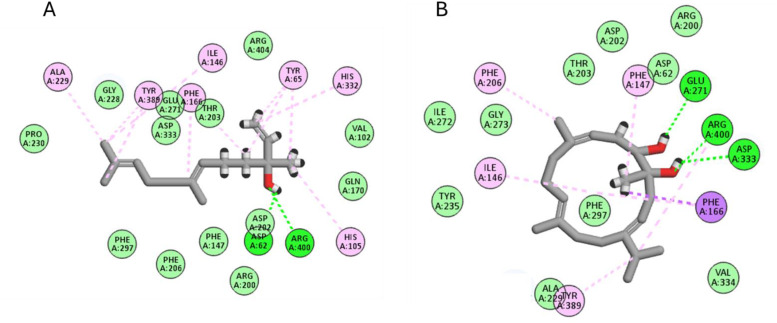
The docking of (A) nerolidol, (B) (1*S*,2*E*,4*S*,5*R*,7*E*,11*E*)-cembra-2,7,11-trien-4,5-diol, in the active site of*α*-glucosidase enzyme (PDB code: 3wy2).

For *α*-glucosidase, the six studied compounds achieved excellent docking scores from −7.9 to −12.3 Kcal/mol. Nerolidol and (1*S*,2*E*,4*S*,5*R*,7*E*,11*E*)-cembra-2,7,11-trien-4,5-diol achieved docking scores of −12.3 and −10.2 Kcal/mol, respectively, ranking the best two compounds. Inspecting their interactions as shown in Fig 8, it was found that nerolidol interacted with Asp62, Tyr65, His105, Ile146, Phe166, Ala229, His332, Tyr389, and Arg400 through different hydrophobic and hydrogen bond interactions. Similarly, (1*S*,2*E*,4*S*,5*R*,7*E*,11*E*)-cembra-2,7,11-trien-4,5-diol interacted with Ile146, Phe147, Phe166, Phe206, Glu271, Asp333, Tyr389 and Arg400.

## 4. Discussion

Here, the essential oil and *n*-hexane extract of *B. serrata* were analyzed *via* GC/MS to identify and quantify their volatile components. As discussed in the results section, about 62 and 71 compounds were identified in the essential oil and *n*-hexane extract.

*Boswellia serrata,* also known as frankincense or olibanum, is widely used for the treatment of various medical conditions, *viz*., arthritis, rhinitis, asthma, and several cancers. Upon reviewing the literature on *B. serrata*, one study reported the in vitro anti-osteoarthritis activity of olebanum gum resin ethanol extract with its main components (keto-*β*-boswellic acid and 3-*O*-acetyl-11-keto-*β*-boswellic acid) [47]. Another study discussed the potential anti-inflammatory activity of *Boswellia serrata* in vitro using ELISA. The oleogum resin was extracted with *n*-hexane, where it was found to be rich with polysaccharides, mucilage, and proteins in a percentage of 35.91%, 34%, and 14.29%, respectively, and the extract showed 82% inhibition of IL-6 [48].

Another species of *Boswellia,* known as *B. sacra* (*B. carterii*) was evaluated as an antiepileptic agent through zebrafish and mouse epilepsy models. Different extracts were prepared, but the n-hexane extract presented the most potent antiepileptic activity. The *n*-hexane extract showed the presence of different triterpenes, including prenyl-*bi*-cyclo-germacrene derivative, *β*-boswellic acid, bosgermacrene A, 11-keto-*β*-boswellic acid, 3-*O*-acetyl-11-keto-*β*-boswellic acid, *α*-boswellic acid, 3-*O*-acetyl *α*-boswellic acid, and 3-*O*-acetyl *β*-boswellic acid [49].

*B. serrata n*-hexane was assessed as anti-inflammatory in the osteoarthritis model (collagen-induced arthritis) in rats. Biochemical markers such as lipid peroxidase, glutathione, catalase, superoxide dismutase and nitric oxide were tested together with inflammatory markers such as IL-1*β*, IL-6, TNF-*α*, IL-10, and IF-*γ* using ELISA [50,51].

Incensole is identified as the main potential component in *Boswellia* species, known for its strong anti-inflammatory properties. The content of incensole in three *Boswellia* species, namely *B. papyrifera*, *B. sacra*, and *B. serrata* was analyzed using HPLC. *B. papyrifera* resin methanol extract had the highest concentration of incensole (18.4%), followed by *n*-hexane (13.5%) and ethyl acetate (3.6%). At the same time, only trace amounts were found in the *B. sacra* fraction, and incensole was not detected in the fractions of *B. serrata* [52]. It is worth noting here that icensole was detected herein in our study in the *n*-hexane extract evaluated through GC/MS analysis, with a percentage of 28.56% and the oxidized derivative named icensole oxide (1.37%).

*B. serrata* oleogum resin was tested in vitro for potential anticancer activity against HepG_2_ and HCT 116 cancer cell lines. GC/MS analysis was performed for the petroleum ether extract of the oleogum resin. The main identified volatile constituents were tricosane (75.32%), sabinene (19.11%), terpinen-4-ol (14.64%), and terpinyl acetate (13.01%) together with cholesterol, stigmasterol, and *ß*-sitosterol as minor components. The petroleum ether extract showed a potent anticancer effect (IC_50_ = 5.82 *μ*g/mL at 48 h) compared to doxorubicin (IC_50_ = 4.68 *μ*g/mL at 48 h) for the HepG_2_ cell line. Regarding the HCT 116 cell line, the IC_50_ value was 6.59 *μ*g/mL at 48 h compared to 5-fluorouracil (IC_50_ = 3.43 *μ*g/mL at 48 h) [11].

Moreover, the n-hexane extract of the oleogum resin from *B. serrata* was assessed for its hepatoprotective effects against liver injuries induced by CCl_4_, paracetamol, and thioacetamide. Silymarin was used as the reference standard. The *n*-hexane extract notably decreased the elevated serum marker enzyme level and prevented the increase in liver weight in all three liver injury models [53]. The antioxidant potential of the hydrodistilled oils and n-hexane extracts were investigated using various methods. A single antioxidant test is insufficient to capture the complex, multifaceted mechanisms by which natural extracts exert antioxidant activity. Therefore, we selected a panel of assays that target different modes of action to ensure a more comprehensive evaluation. In particular, we utilized radical scavenging assays like DPPH and ABTS, as they assess the capacity of compounds to donate hydrogen atoms or electrons, neutralizing free radicals, and demonstrating direct radical-quenching abilities. We included reducing power assays such as CUPRAC and FRAP to assess the electron-donating capabilities of the extracts, a crucial aspect of antioxidant defense. Additionally, we employed the metal-chelating assay to assess the ability to bind transition metals like Fe² ⁺ , thereby preventing Fenton-type reactions that produce highly reactive hydroxyl radicals. The phosphomolybdenum assay was used to determine total antioxidant capacity, offering a comprehensive index of both water- and lipid-soluble antioxidant compounds.

In general, essential oils showed greater abilities among the methods. The *n*-hexane extract was only more active than the essential oil in the FRAP test. The observed antioxidant effect of essential oils can be explained by the presence of some volatile compounds. For example, the essential oil contained nerolidol and was described in previous work as an important antioxidant [54,55]. In addition to nerolidol, acetic acid octyl ester, *α*-pinene, and incensole may contribute to the observed antioxidant properties. However, because the *n*-hexane extract was richer in hydrocarbons than the essential oil, it showed lower antioxidant potential. In the literature, the essential oil of *B. serrata* gum has remarkable antioxidant properties. For example, a study by Gupta et al (2017) [56] examined the chemical composition of *B. serrata* essential oils for antioxidant properties, and they exhibited greater radical scavenging ability in the DPPH assay at a 100 *µ*g/ml concentration. In another study by Irahal et al (2021), the essential oil *B. serrata* exhibited stronger protection against lipid peroxidation in the *β*-carotene/linoleic acid test system [57]. The essential oil of *B. serrata* also exhibited stronger radical scavenger ability than BHT in the DPPH assay, as reported by [58]. Similar results were also reported by [59].

Enzymes act as catalysts, speeding up many biochemical reactions. Their function is influenced by their unique structural shapes within biological systems. Enzyme inhibition occurs when an inhibitor molecule reduces or stops enzyme activity. This happens when the inhibitor interferes with the enzyme’s ability to bind to its natural substrate, thereby limiting the production of products.

In this study, we evaluated the inhibitory effects of the essential oil and *n*-hexane extract of *B. serrata* against several key enzymes. We selected the enzymes because they are associated with global health problems. For example, inhibiting AChE can increase the level of acetylcholine in the synaptic cleft, which can improve the cognitive function of Alzheimer’s patients. This phenomenon is also known as the cholinergic hypothesis and forms the basis of the main strategy for developing Alzheimer’s drugs [60]. In addition, amylase and glucosidase are the main enzymes involved in the hydrolysis of carbohydrates, and their inhibition can help to manage the blood glucose levels of diabetic patients [61]. Tyrosinase is the main enzyme involved in melanin synthesis, and thus its inhibition can address hyperpigmentation issues [62]. These disorders affect people all over the world, which is why we aim to provide a novel approach to managing them via the theory of enzyme inhibition.

Consistent with the antioxidant results, except for amylase inhibition, the essential oil showed stronger inhibitory effects than the *n*-hexane extract. Some constituents in essential oil can contribute to these abilities. For example, nerolidol has been reported as a significant AChE inhibitor in a previous study [63]. In addition, several studies have reported that nerolidol has a good neuroprotective effect. Furthermore, incensole prevented the formation of *β*-amyloid plaque in Alzheimer’s disease and thus can be considered a neuromodulator agent [64]. *Alpha*-pinene is a good anti-cholinesterase inhibitor in several studies [65]. In a previous study by Wu et al. (2020), cembran-type diterpenoids were reported as novel glucosidase inhibitors. Overall, *B. serrata* essential oil in particular may be a useful active ingredient in the production of novel drugs to address global health issues like diabetes and Alzheimer’s disease [66].

Understanding the molecular mechanism of compounds provides a futuristic guide for further drug development and optimization. Accordingly, five targets commonly found in many chronic diseases were selected as potential targets for docking of the compounds in the major extract. The docking results revealed the excellent ability of many compounds in the major extract to strongly bind and inhibit the selected targets. To this end, the docking results highlight the Hexane Extract and the Hydrodistilled Oil of *Boswellia serrata* as potential source for finding a cure for many diseases.

## 5. Conclusions

GC–MS analysis of the essential oil and *n*-hexane extract of *Boswellia* (olibanum oleogum) revealed a diverse profile of volatile terpenoids and related compounds. The essential oil exhibited significantly higher antioxidant and enzyme inhibitory activities than the *n*-hexane extract, indicating that its bioactive constituents play a major role in modulating oxidative and enzymatic pathways. These findings support the potential therapeutic value of olibanum essential oil in managing oxidative stress-related and age-associated disorders, including Alzheimer’s disease, *Diabetes mellitus*, and skin hyperpigmentation. This study provides scientific evidence for the traditional use of frankincense and highlights its potential as a source of natural compounds for drug and cosmetic development. Future investigations should aim to isolate and characterize the key active constituents, elucidate their molecular mechanisms, and assess their safety, pharmacokinetics, and efficacy through *in vivo* and clinical studies. Such work will be essential to fully establish the therapeutic relevance and translational potential of olibanum essential oil.
