# Correction: Comparative metabolic profiling, enzyme inhibitory activities, and *in-silico* analysis of the hexane extract and the hydrodistilled oil of *Boswellia serrata*

**DOI:** 10.1371/journal.pone.0352773

**Published:** 2026-06-26

**Authors:** Heba A. S. El-Nashar, Esraa A. Elhawary, Nilofar Nilofar, Mahmoud A. El Hassab, Taghreed A. Majrashi, Wagdy Eldehna, Gokhan Zengin, Omayma A. Eldahshan

The Funding statement for this article is incorrect. The correct Funding statement is as follows: The authors extend their appreciation to the Deanship of Research and Graduate Studies at King Khalid University for funding this work through Large Research Project under grant number (RGP2/500/46).

## References

[1] El-Nashar HAS, Elhawary EA, Nilofar N, El Hassab MA, Majrashi TA, Eldehna W, et al. Comparative metabolic profiling, enzyme inhibitory activities, and *in-silico* analysis of the hexane extract and the hydrodistilled oil of *Boswellia serrata*. PLoS One. 2026;21(5):e0348178. doi: 10.1371/journal.pone.0348178 42102034 PMC13155625

